# Targeted therapy and immunotherapy for gastric cancer: rational strategies, novel advancements, challenges, and future perspectives

**DOI:** 10.1186/s10020-025-01075-y

**Published:** 2025-02-08

**Authors:** Dong Luo, Yunmei Liu, Zhengmao Lu, Lei Huang

**Affiliations:** 1 Department of Gastroenterology, National Clinical Research Center for Digestive Diseases, The First Affiliated Hospital of Naval Medical University/Changhai Hospital, Naval Medical University, 168 Changhai Road, Shanghai, 200433 China; 2 School of Cultural Heritage and Information Management, Shanghai University, Shanghai, 200444 China; 3 Department of Gastrointestinal Surgery, The First Affiliated Hospital of Naval Medical University, Shanghai, 200433 China; 4 National Key Laboratory of Immunity and Inflammation, Changhai Clinical Research Unit, The First Affiliated Hospital of Naval Medical University/Changhai Hospital, Naval Medical University, 168 Changhai Road, Shanghai, 200433 China; 5 Center of Structural Heart Disease, Fuwai Hospital, Chinese Academy of Medical Sciences and Peking Union Medical College, Beijing, China

**Keywords:** Gastric cancer, Targeted therapy, Immunotherapy

## Abstract

Gastric cancer (GC) is one of the most common malignant tumors worldwide, and its treatment has been a focus of medical research. Herein we systematically review the current status of and advancements in targeted therapy and immunotherapy for GC, which have emerged as important treatment strategies in recent years with great potential, and summarize the efficacy and safety of such treatments. Targeted therapies against key targets in GC, including epidermal growth factor receptor (EGFR), human epidermal growth factor receptor 2 (HER2), and vascular endothelial growth factor (VEGF)/VEGF receptor (VEGFR), have shown remarkable therapeutic efficacies by inhibiting tumor progression and/or blood supply. In particular, markable breakthroughs have been made in HER2-targeting drugs for HER2-positive GC patients. To address intrinsic and acquired resistances to HER2-targeting drugs, novel therapeutic agents including bispecific antibodies and antibody–drug conjugates (ADC) targeting HER2 have been developed. Immunotherapy enhances the recognition and elimination of cancer cells by activating body anticancer immune system. Programmed cell death protein 1 (PD-1) and programmed cell death-ligand 1 (PD-L1) antibodies are the most commonly used immunotherapeutic agents and have been used with some success in GC treatment. Innovative immunotherapy modalities, including adoptive immune cell therapy, tumor vaccines, and non-specific immunomodulators therapy, and oncolytic viruses have shown promise in early-stage clinical trials for GC. Clinical trials have supported that targeted therapy and immunotherapy can significantly improve the survival and quality of life of GC patients. However, the effects of such therapies need to be further improved and more personalized, with advancement in researches on tumor immune microenvironment. Further studies remain needed to address the issues of drug resistance and adverse events pertaining to such therapies for GC. The combined application of such therapies and individualized treatment strategies should be further explored with novel drugs developed, to provide more effective treatments for GC patients.

## Introduction

According to Global Cancer Statistics 2022 by the WHO, gastric cancer (GC) is the fifth most common type of malignancy and the fifth leading cause of cancer-related deaths globally (Sung et al. 2021; Huang et al. 2022a, b). The majority of GC patients are diagnosed at an advanced stage with a poor prognosis due to the lack of typical clinical presentations (Qiu et al. 2021; Huang et al. 2017, 2013, 2020a). Traditional treatments for GC mainly include resection, chemotherapy, and radiotherapy (Huang et al. 2020b, c; Wei et al. 2020a). Resection is the mainstay treatment for GC, but its application is limited for late-stage GC (Huang and Li 2018; Xu et al. 2014a, b; Huang et al. 2019). Chemotherapy and radiotherapy can be used before and/or after surgery, but their efficacies are limited and largely heterogeneous, often accompanied by a series of adverse events (Xu et al. 2014c; Huang et al. 2014a; Huang and Xu 2014). With in-depth researches on the mechanism of GC, targeted therapy and immunotherapy have become hot topics (Meng et al. 2020).

Targeted therapy can accurately identify specific markers and attack cancer cells causing less damage to normal cells (Chen et al. 2018; Huang et al. 2023a). It has the advantages including improved therapeutic effects and reduced adverse events, and has become a hot spot for individualized treatment of GC. Representatively, trastuzumab combined with chemotherapy prolonged overall survival (OS) by 2.7 months compared with chemotherapy alone in the ToGA study (Bang et al. 2010), after which, however, progress in the development of therapeutic agents against GC stalled for nearly a decade. Although drugs targeting epidermal growth factor receptor (EGFR), human epidermal growth factor receptor 2 (HER2), and vascular endothelial growth factor (VEGF)/VEGF receptor (VEGFR) has also been under development during this period, none of them had a very favorable efficacy. This state of affairs has recently been broken with the advents of novel anti-HER2 agents and immunotherapy for GC. Novel anti-HER2 therapeutics (e.g., trastuzumab deruxtecan (T-DXd; DS-8201a) and disitamab vedotin (RC48)) have made significant breakthroughs in the treatment of GC (Nakamura et al. 2021). Immunotherapy activates patients’ own anticancer immune system with enhanced and long-lasting immune responses against cancer cells, and effectively prolongs the survival of GC patients (Jiang et al. 2019). Specifically, anti-programmed cell death protein 1 (PD-1) antibodies have shown more durable anticancer immunity and longer survival in untreated metastatic GC patients with highly unstable microsatellites (microsatellite instability-high (MSI-H)/deficient mismatch repair (dMMR)) (Chao et al. 2021). Emerging immunotherapies such as adoptive immune cell therapy, tumor vaccines, and non-specific immunomodulators therapy, and oncolytic viruses have brought more personalized and comprehensive treatment options for GC. The combined application of targeted therapy and immunotherapy may be a future research direction to further improve the efficacy of GC treatment.

Herein we review the current status and progress of targeted therapy and immunotherapy for GC, highlighting the mechanisms of action of relevant drugs and systematically summarizing the relevant clinical trials, and provide perspectives into the development trends of the treatment in the future.

## Targeted therapies for GC

With in-depth researches on the molecular mechanism of GC, targeted therapy plays an increasingly important role in the individualized and precise treatment of GC. Targeted therapy mainly interferes with malignant behaviors by inhibiting key signaling pathways. In GC, common targeted therapy targets include EGFR/HER2 (Wei et al. 2020b) and VEGF/VEGFR (Table 1).

**Table 1:** Clinical trials of targeted therapy

Type	Drugs	Clinical trials	Research objects	Regimens	Conclusion of major findings	Adverse event (AE)
EGFR-targeting drugs	Cetuximab	Phase II trial: NCT00477711 (Zhang et al. 2014)	Unresectable or metastatic GC or GEJA	Cetuximab in combination with cisplatin and capecitabine	Cetuximab in combination with cisplatin and capecitabine was efficacious and well tolerated as first-line treatment for AGC or EGJA	Most common AEs: neutropenia (25.0%), nausea/vomiting (11.5%), and rash/exfoliation (9.6%)
	Panitumumab	Phase II trial: NCT01379807 (Quintero Aldana et al. 2020)	Advanced or unresectable GC or GEJA	Panitumumab in combination with docetaxel and cisplatin	Adding panitumumab to standard chemotherapy as first-line treatment did not improve efficacy outcomes	Most common AEs: weakness (75.0%) and mucosal inflammation (54.5%)
	GC1118	Phase I trial: NCT02352571 (Oh et al. 2019)	Refractory solid cancers (GC and colorectal cancer)	GC1118	GC1118 showed good anticancer activity and was well tolerated	Most common AE: skin toxicity
Her2-targeting drugs	Trastuzumab	Phase II trial TOGA: NCT01041404 (Bang et al. 2010)	HER2-positive AGC or GEJA	Trastuzumab + chemotherapy vs chemotherapy alone	Trastuzumab in combination with chemotherapy emerged as a first-line treatment option for HER2-positive AGC	Most common AEs: vomiting (50% vs 46%) and neutropenia (53% vs 57%)
	Pertuzumab	Phase II trial JACOB: NCT01774786 (Tabernero et al. 2018)	HER2-positive metastatic GC or GEJA	Pertuzumab + trastuzumab + chemotherapy vs placebo + trastuzumab + chemotherapy	Pertuzumab + trastuzumab + chemotherapy as first-line treatment for HER2-positive metastatic GC or GEJA did not significantly improve OS compared with standard of care	Most common grade 3–5 AEs: neutropenia (30% vs 28%), anemia (15% vs 17%), and diarrhea (13% vs 6%)
	Lapatinib	Phase II trial S0413: NCT00103324 (Iqbal et al. 2011)	AGC or metastatic GC	Lapatinib	Lapatinib had modest single agent activity for advanced/metastatic GC	Most common grade 3–4 AEs: fatigue, anorexia, and diarrhea
		Phase III trial TRIO-013/LOGIC: NCT00680901 (Hecht et al. 2016)	HER2-amplified advanced GEJA	Lapatinib + capecitabine and oxaliplatin (CapeOx) vs placebo + CapeOx	Addition of lapatinib to capecitabine and oxaliplatin did not significantly increase OS in HER2-amplified GEJA	Most common grade 3–5 AEs: diarrhea (58% vs 29%), nausea (49% vs 43%), vomiting (44% vs 36%), and decreased appetite (41% vs 32%)
ADC	T-DM1	Phase II/III trial GATSBY: NCT01641939 (Thuss-Patience et al. 2017)	Previously treated HER2-positive locally advanced or metastatic GC or GEJA	T-DM1 vs taxane	T-DM1 was not superior to paclitaxel analogs in previously-treated HER2-positive AGC	Most common grade 3–5 AEs: anemia (26%) and thrombocytopenia (11%) in the T-DM1 group and neutropenia (39%) and anemia (18%) in the paclitaxel group
	T-DXd	Phase I trial (Doi et al. 2017)	Advanced stomach or gastroesophageal and breast cancers	T-DXd	The most likely recommended phase 2 dosing was 5.4 or 6.4 mg/kg	Most common grade 3 AEs: lymphopenia and decreased neutrophil count
		Phase I trial: NCT02564900 (Shitara et al. 2019)	HER2-positive AGC	T-DXd	T-DXd showed a manageable safety profile and preliminary activity in HER2-positive GC or GEJA	Most common grade 3–5 AEs: anemia (30%) and declines in neutrophils (20%), platelets (18%), and white blood cell counts (16%)
		Phase II trial DESTINY-Gastric01: NCT03329690 (Shitara et al. 2020a)	HER2-positive AGC	T-DXd vs chemotherapy	T-DXd significantly improved response and OS in HER2-positive GC compared to standard therapy	Most common grade 3–5 AEs: decreased neutrophil count (51% vs 24%), anemia (38% vs 23%), and decreased white blood cell count (21% vs 11%)
		Phase II trial DESTINY-Gastric02: NCT04014075 (Cutsem et al. 2021)	AGC and GEJA	T-DXd	T-DXd was effective in unresectable or metastatic HER2-positive GC or GEJA	Most common grade 3 + AEs: anemia (14%), nausea (8%), decreased neutrophil count (68%), and decreased white blood cell count (6%)
	RC48	Phase I trial: NCT02881190 (Xu et al. 2021)	HER2-positive solid tumors (especially GC)	RC48	RC48 was well tolerated in HER2-positive solid tumors and showed promising antitumor activity	Most common grade 3 + AEs: neutropenia (19.3%), leukopenia (17.5%), hyperalgesia (14.0%), and elevated bound blood bilirubin (8.8%)
		Phase II trial: NCT03556345 (Peng et al. 2021)	HER2-overexpressing AGC or GEJA with ≥ 2 prior lines of chemotherapy	RC48	RC48 showed promising activity and a manageable safety profile	Most common AEs: decreased white blood cell count (53.6%), weakness (53.6%), alopecia (53.6%), decreased neutrophil count (52.0%), anemia (49.6%), and increased aspartate aminotransferase levels (43.2%)
Bispecific antibodies	ZW25	Phase I trial: NCT02892123 (Meric-Bernstam et al. 2021)	GEJA expressing HER2	ZW25 vs ZW25 + chemotherapy	ZW25 was well tolerated in previously treated GEJA and showed durable anticancer activity	The most common AEs: ZW25, diarrhea (44%) and infusion-related reactions (36%); ZW25 + chemotherapy, diarrhea (58%) and fatigue (27%)
		Phase II trial: NCT03929666 (Ku et al. 2021)	Advanced/metastatic HER2-expressing GEJA	ZW25 + chemotherapy (mFOLFOX6, CAPOX, or 5-FU/FP)	ZW25 was well tolerated and had a long-lasting response	Most common AEs: diarrhea, nausea, peripheral sensory neuropathy, fatigue, decreased appetite, vomiting, and hypokalemia
	KN026	Phase II trial: NCT03925974 (Xu et al. 2023)	Previously treated AGC or GEJA expressing HER2	KN026	KN026 showed a favorable safety profile and promising antitumor activity	Most common grade 3 + AEs: gastrointestinal disorders (11%)
VEGF/VEGFR-targeting drugs	Bevacizumab	Phase III trial AVAGAST: NCT00548548 (Ohtsu et al. 2011)	AGC	Bevacizumab + fluoropyrimidine-cisplatin vs placebo + fluoropyrimidine-cisplatin	The addition of bevacizumab to chemotherapy was associated with a significant increase in PFS and ORR as first-line treatment of advanced GC	Most common grade 3–5 AEs: neutropenia (35% vs 37%), anemia (10% vs 14%), and decreased appetite (8% vs 11%)
	Ramucirumab	Phase III trial REGARD: NCT00917384 (Fuchs et al. 2014)	AGC or GEJA which progressed after first-line chemotherapy	Ramucirumab vs placebo	Ramucirumab had a survival benefit in AGC or GEJA which progressed after first-line chemotherapy	Most common grade 3 + AEs: hypertension (16% vs 8%) and bleeding (13% vs 11%)
		Phase III trial RAINBOW: NCT01170663 (Wilke et al. 2014)	Previously treated AGC or GEJA	Ramucirumab + paclitaxel vs placebo + paclitaxel	Compared with placebo + paclitaxel, ramucirumab + paclitaxel significantly improved OS and could be considered a new standard second-line treatment for patients with advanced GC	Most common grade 3 + AEs: neutropenia (41% vs 19%), leukopenia (17% vs 7%), hypertension (14% vs 2%), fatigue (12% vs 5%), anemia (9% vs 10%), and abdominal pain (6% vs 3%)
		Phase II trial: NCT01246960 (Yoon et al. 2016)	Advanced gastric adenocarcinoma, GEJA, or esophageal cancer	mFOLFOX6 + ramucirumab vs mFOLFOX6 + placebo	Addition of ramucirumab to first-line mFOLFOX6 did not significantly improve PFS	Most common grade 3 AEs: neutropenia (26.8% vs 36.3%), fatigue (18.3% vs 15.0%), and hypertension (15.9% vs 3.8%)
		Phase III trial RAINFALL: NCT02314117 (Fuchs et al. 2019)	Metastatic GC or GEJA	Ramucirumab + fluoropyrimidine + cisplatin vs ramucirumab + fluoropyrimidine + cisplatin	The addition of ramucirumab to cisplatin in combination with fluoropyrimidine chemotherapy was not recommended as first-line therapy for metastatic GC or GEJA	Most common grade 3–4 AEs: neutropenia (26% vs 27%), anemia (12% vs 14%), and hypertension (10% vs 2%)
	Apatinib	Phase III trial (Li et al. 2016)	Advanced or metastatic stomach adenocarcinoma or GEJA	Apatinib vs placebo	Apatinib significantly improved OS and PFS with an acceptable safety profile in patients with advanced GC refractory to two or more prior chemotherapies	Most common AEs: leukopenia (40.3% vs 8.8%), neutropenia (37.5% vs 9.9%), anemia (25.0% vs 24.2%)

*AGC* advanced gastric cancer, *GEJA* gastroesophageal junction adenocarcinoma, *OS* overall survival, *PFS* progression-free survival, *AE* adverse event

### EGFR-targeting drugs for GC

The EGFR family belongs to the receptor tyrosine kinase (RTK) family and possesses both tyrosine kinase activity and ligand binding capacity. The family includes four members: EGFR (HER1), HER2, HER3, and HER4. Upon activation, EGFR promotes malignant behaviors by signaling into the nucleus and activating downstream signaling pathways. Overexpression of EGFR is often associated with high aggressiveness and low survival in GC (Dulak et al. 2012). Drugs targeting EGFR can inhibit the proliferation, invasion, and migration of cancer cells by blocking relevant signaling pathways to treat GC (Adashek et al. 2020). Main EGFR-targeting drugs include cetuximab, panitumumab, and GC1118.

Cetuximab is a human-mouse chimeric lgG1 monoclonal antibody (mAb) against EGFR, which specifically binds to EGFR and blocks the intracellular signaling pathway through the inhibition of tyrosine kinase binding to EGFR, thereby inhibiting the proliferation of cancer cells and inducing apoptosis (Raimúndez et al. 2020; Oda et al. 2005). Several phase II trials (Pinto et al. 2007; Han et al. 2009; Kim et al. 2011) suggested that cetuximab in combination with chemotherapy was effective in treating advanced GC (AGC). However, the phase III EXPAND trial (Lordick et al. 2013) suggested that the addition of cetuximab to capecitabine plus cisplatin had no additional benefit over chemotherapy alone as first-line treatment for AGC. Notably, in this trial EGFR expression was not tested. A multicenter phase II trial (NCT00477711) (Zhang et al. 2014) showed that patients receiving cetuximab plus cisplatin/capecitabine (C + XP) had an objective remission rate (ORR) of 53.2%, a median progression-free survival (mPFS) of 5.2 months, and a median overall survival (mOS) of 10.8 months, suggesting the good efficacy and tolerability of the combination as first-line treatment for AGC or gastroesophageal junction adenocarcinoma (GEJA). EGFR overexpression might predict the efficacy of cetuximab treatment.

Panitumumab is a fully humanized lgG2 mAb against EGFR, and has the advantage of minimal immunogenicity. While panitumumab has been approved for the treatment of patients with EGFR-positive colorectal cancer resistant to standard therapy (Cutsem et al. 2007), it has not fared well in the treatment of GC. The phase III REAL3 trial (Waddell et al. 2013) demonstrated that the addition of panitumumab to epirubicin, oxaliplatin, and capecitabine (EOC) chemotherapy did not significantly increase OS, and was not recommended for unselected patients with advanced esophagogastric adenocarcinoma. Given the unselected population of the REAL3 trial, a clinical trial on anti-EGFR therapy for EGFR-amplified gastroesophageal adenocarcinoma was conducted, which showed a high response rate and a long survival period (Maron et al. 2018). However, a phase II trial showed that adding panitumumab to standard chemotherapy (docetaxel and cisplatin) as first-line treatment did not significantly improve the efficacy outcomes in patients with AGC or GEJA (Quintero Aldana et al. 2020).

GC1118 is a novel fully humanized anti-EGFR mAb that binds to the domain III epitope of EGFR at the N-terminal, without overlapping the epitopes of other EGFR-targeting antibodies. Compared with cetuximab and panitumumab, GC1118 exhibits excellent EGFR binding and inhibitory activities against high-affinity EGFR ligands, inhibiting EGFR signaling and proliferation (Lim et al. 2016). A preclinical study (Park et al. 2019) showed that GC1118 exerted a more potent antitumor effect against GC cells when used alone or in combination with cytotoxic chemotherapeutic agents compared to cetuximab. The first-in-human phase I study of GC1118 demonstrated that GC1118 had good anticancer activity and tolerability in patients with refractory solid tumors (Oh et al. 2019). A phase II trial evaluating GC-1118 in combination with paclitaxel as second-line treatment for GC and GEJA (NCT04077255) is ongoing in Korea.

Together, mAbs against EGFR including cetuximab and panitumumab have shown some efficacies in treating GC of different stages, but the findings of some clinical trials have not been favorable. Precise subject selection based on predictors including EGFR overexpression may help to improve the efficacies of these mAbs in specific patient populations. The novel anti-EGFR mAb GC1118 demonstrated stronger antitumor effects against GC cells, and its potential as treatment for GC deserves further in-depth exploration.

### HER2-targeting drugs for GC

HER2 is the first meaningful target identified in the history of GC treatment. HER2 is a transmembrane RTK which lacks specific ligands and which transmits cell proliferation signals by forming heterodimers with other HER family members, leading to phosphorylation of tyrosine residues and triggering downstream signaling cascades that regulate cancer cell survival, proliferation, apoptosis, differentiation, motility, adhesion, invasion, and migration, and tumor angiogenesis (Yarden and Sliwkowski 2001; Guo et al. 2023). Main anti-HER2 drugs include trastuzumab, pertuzumab, and lapatinib.

#### HER2-targeting mAb

Trastuzumab is a humanized recombinant IgG1 mAb, which directly targets the extracellular region of the HER2 receptor, and it can block and cleave the receptor inhibiting HER2 heterodimerization (Guo et al. 2021a). Consequently, it blocks HER2-mediated signaling and promotes antibody-dependent cytotoxicity, causing death of HER2-expressing GC cells (Namboodiri and Pandey 2011). In 2010, the ToGA study (Bang et al. 2010) showed that the OS was 13.8 (95% CI = 12–16) months in the trastuzumab-chemotherapy group and 11.1 (95% CI = 10–13) months in the chemotherapy group (HR = 0.74; 95% CI = 0.60–0.91; *P* = 0.005), corresponding to a 26% reduction in mortality. At present, trastuzumab combined with chemotherapy has become the first-line treatment option for HER2-positive advanced GC. The latest Chinese Society of Clinical Oncology (CSCO), the Japanese Gastric Cancer Association (JGCA), and the National Comprehensive Cancer Network (NCCN) guidelines all recommend such combination as the first-line treatment for HER2-positive metastatic gastric adenocarcinoma (Japanese Gastric Cancer Association 2021; Zhang et al. 2023; Huang et al. 2022c).

Pertuzumab is a humanized mAb which inhibits HER2 dimerization by preventing the pairing of HER2 with other EGFR receptors (including HER3), thereby inhibiting downstream signaling associated with tumor growth and progression. In contrast, trastuzumab does not inhibit HER2-HER3 interactions. Pertuzumab binds to an epitope in the extracellular dimerization domain II of HER2, different from the site where trastuzumab binds to (epitope in domain IV) (Scheuer et al. 2009; Baselga and Swain 2009). In 2014, the phase IIa JOSHUA trial reported higher tumor remission rates when combining higher doses of pertuzumab (840 mg) with chemotherapy. In safety assessment, diarrhea rate was higher in the combination group. The subsequent phase III JACOB trial in 2018 demonstrated that the addition of pertuzumab (840 mg every three weeks) to trastuzumab and chemotherapy as first-line treatment for HER2-positive metastatic GC or GEJA tended to increase OS compared with standard treatment (17.5 vs 14.2 months, HR = 0.84, *P* = 0.057) (Kang et al. 2014a; Tabernero et al. 2018; Huang et al. 2022d).

#### HER2-targeting tyrosine kinase inhibitors (TKI)

TKI inhibits the activity of tyrosine kinases, primarily barring the phosphorylation of protein tyrosine residues, blocking downstream signaling pathways, and inhibiting tumor growth and metastasis. Lapatinib can selectively inhibit EGFR (HER1) and HER2 in the intracellular tyrosine kinase domain, and can be used to treat trastuzumab-resistant advanced or metastatic breast cancer (Konecny et al. 2006). In 2011, the Southwest Oncology Group (SWOG) conducted a phase II trial where lapatinib was administered orally at a dose of 1500 mg/day for 28 days to patients with chemotherapy-treated advanced/metastatic GC, and was well tolerated with modest single-agent activity (Iqbal et al. 2011). Notably, the phase III LOGIC trial demonstrated that the addition of lapatinib to chemotherapy (capecitabine and oxaliplatin) did not significantly increase the OS of patients with HER2-amplified advanced gastroesophageal adenocarcinomas, but that lapatinib increased the toxic effects (e.g., diarrhea and skin rash) (Hecht et al. 2016). Therefore, lapatinib was not recommended for GC. Tucatinib is a highly-selective HER2-targeted TKI approved by the Food and Drug Administration (FDA) in 2020 for HER2-positive metastatic breast cancer (Lee 2020), and is currently being explored in GC.

Together, following trastuzumab which significantly improves the prognosis of patients with HER2-positive GC, attempts on improving HER2-targeting therapy for advanced GC have not been as successful as expected. Further studies on HER2-targeting mAb and TKI with improved patient survival and quality of life are needed in different patient subpopulations.

#### Emerging HER2-targeting drugs for GC

The development of HER2-targeting therapies for GC has been hampered by the heterogeneity of HER2 expression and race, and the intrinsic and/or acquired resistance to HER2-targeting drugs in GC, and there is unmet need for drugs with targets beyond the HER2 pathway and with strong antitumor effects. Recently, the following novel HER2-targeted drugs for advanced or metastatic GC have emerged: antibody–drug conjugates (ADC) and bispecific antibodies (Huang and Shi 2022).

#### ADC

Trastuzumab emtansine (T-DM1) is an ADC consisting of trastuzumab and DM1, which interferes with mitosis and promotes apoptosis. Trastuzumab delivers DM1 to cancer cells by binding to the HER2 receptor on the surface of cancer cells. DM1, when released in cancer cells, inhibits microtubule protein synthesis and polymerization, thus blocking the growth signaling pathway of cancer cells, and ultimately leading to cancer cell death. This makes T-DM1 possess both targeted and cytotoxic anticancer effects (Lewis Phillips et al. 2008). In the phase III EMILIA trial (Verma et al. 2012), the T-DM1 group had a higher survival than the lapatinib combined with capecitabine group with lower toxicity, and T-DM1 significantly prolonged the OS and progression-free survival (PFS) in patients with HER2-positive advanced breast cancer who had been previously treated with trastuzumab and paclitaxel analogues. In the phase II/III adaptive GATSBY randomized trial (Thuss-Patience et al. 2017) examining the efficacy and tolerability of T-MD1, mOS was 7.9 months in the T-DM1 group and 8.6 months in the taxane group (HR = 1.15; one-sided *P* = 0.86), and mPFS was 2.7 months in the T-DM1 group and 2.9 months in the taxane group (HR = 1.13; *P* = 0.31). T-DM1 was not superior to paclitaxel analogs in patients previously treated for HER2-positive advanced GC, but with a higher incidence of grade 3 or higher pulmonary toxicity, thrombocytopenia, and bleeding.

T-DXd is a novel ADC consisting of a humanized anti-HER2 antibody, a newly-developed enzymatically-cleavable peptide linker, and a novel potent exatecan derivative topoisomerase I inhibitor with a special structure that reduces the hydrophobicity of the ADC, and can carry eight DXd molecules per antibody (more than other ADC). These unique properties contribute to its preclinical efficacy against T-DM1-insensitive cancers with low HER2 expression (Takegawa et al. 2017). On January 15, 2021, the US FDA approved T-DXd for use in patients with locally-advanced or metastatic HER2-positive GC or GEJA who have received prior trastuzumab treatment (Mishima and Shitara 2021). A Japan phase I dose-escalation study (Doi et al. 2017) where patients with advanced malignancies (GC/GEJA or breast cancer) received 0.8–8.0 mg/kg T-DXd every 3 weeks showed that the maximum tolerated dose (MTD) was not reached and that the most likely recommended dose for Phase II study was 5.4 or 6.4 mg/kg. Subsequently, a phase I dose-expansion trial (Shitara et al. 2019) was conducted in the US and Japan, and demonstrated that patients with HER2-positive GC or GEJA who received at least one recommended dose of T-DXd had an ORR of 43.2% (95% CI = 28.3%−59.0%), a mPFS of 5.6 months, and a mOS of 12.8 months. Thus, T-DXd had a manageable safety profile and showed promising antitumor activity in patients with HER2-positive GC or GEJA. The phase II DESTINY-Gastric01 trial (Shitara et al. 2020a) conducted in Japan and South Korea showed that in patients with HER2-positive GC who had progressed after two or more prior treatments including trastuzumab, the ORR was significantly higher in the T-DXd group than in the standard chemotherapy group (51% vs 14%, *P* < 0.001). OS was also significantly longer in the T-DXd group than in the standard chemotherapy group (12.5 vs 8.4 months; HR = 0.59; *P* = 0.01). The phase II DESTINY-Gastric02 trial (Cutsem et al. 2021) showed that TDX-d was effective in patients with unresectable or metastatic HER2-positive GC or GEJA in the US or Europe (ORR = 38.0%). In addition, the phase 3 DESTINY-Gastric04 study (NCT04704934) comparing TDX-d with paclitaxel plus ramucirumab in the second-line setting regarding efficacy and the phase Ib/II DESTINY-Gastric03 (NCT04379596) trial evaluating the efficacy of TDX-d in combination with chemotherapy and the immune checkpoint inhibitor pembrolizumab or durvalumab in the first- or second-line setting are ongoing.

RC48 is a novel HER2-targeted ADC drug independently developed in China. It is coupled with a mAb, a cleavable linker, and Methyl Marinol E (MMAE), which can be precisely delivered into cancer cells (Zhu et al. 2021). RC48 employs a new, humanized antibody with higher affinity and better endocytosis, and uses advanced linkers and small molecule toxin drugs to achieve highly efficient killing of cancers. Unlike T-DM1, RC48 has a bypass killing effect on nearby cancer cells regardless of HER2 status, which helps to overcome spatial heterogeneity and enhance anticancer effects. Its use for GC is being tested. A phase I study (Xu et al. 2021) demonstrated that in patients with HER2-positive advanced solid tumors, especially GC with low HER expression RC48 had good antitumor activity. A phase II study (Peng et al. 2021) on RC48 in patients with advanced or metastatic HER2-positive GC or GEJA showed an ORR of 24.8% (95% CI = 17.5–33.3%), with a mPFS and mOS of 4.1 (95% CI = 3.7–4.9) months and 7.9 (95% CI = 6.7–9.9) months, respectively. Based on the findings of this trial, RC48 received conditional marketing approval from the State Food and Drug Administration (SFDA) in June 2021 for the treatment of patients with locally advanced or metastatic GC or GEJA who have received at least two types of chemotherapy. Furthermore, a phase II/III trial (NCT05980481) evaluating the safety and efficacy of RC48 in combination with toripalimab and chemotherapy or RC48 in combination with toripalimab and Herceptin as first-line treatment for patients with locally advanced or metastatic HER2-expressing GC and a phase III trial (NCT04714190) evaluating the efficacy and safety of RC48 for the treatment of HER2-overexpressing locally advanced or metastatic GC are ongoing.

An important challenge with HER2-targeting drugs for GC is the heterogeneity of HER2 (e.g., protein expression variations and gene copy number alterations) within cancers (Zhu et al. 2021). Many GC cases may exhibit varying levels of HER2 positivity, and even within a single tumor, there can be areas that are HER2-positive and others that are HER2-negative (Park et al. 2016; Cho et al. 2013). This intra-tumoral heterogeneity can lead to inconsistent therapeutic responses, making it difficult to predict which patients will benefit most from HER2-targeting therapies. In addition, accurate and standardized testing methods for HER2 status are critical but can vary in practice. Misclassification of HER2 status due to sampling errors and/or differences in testing protocols can lead to inappropriate treatment decisions. These challenges highlight the urgent need for refined biomarker assessment techniques and better understanding of tumor biology to optimize therapeutic strategies for patients with GC.

#### Bispecific antibodies

Zanidatamab (ZW25) is a novel HER2-targeting bispecific antibody that binds to HER2 extracellular domains II and IV, the same domains targeted by trastuzumab and pertuzumab. According to a phase I trial, ZW25 was well tolerated and showed durable anticancer activity in heavily-pretreated gastroesophageal adenocarcinoma (GEA) patients (Meric-Bernstam et al. 2021). First-line ZW25 plus combination chemotherapy in HER2-expressing GEA also showed encouragingly good tolerability, manageable safety profile, and durable response in a multicenter phase II study (Ku et al. 2021). Based on these findings, a global phase III trial was designed to evaluate the efficacy and safety of ZW25 combination chemotherapy with or without tirelizumab compared with standard therapy (trastuzumab in combination with chemotherapy) as first-line treatment of patients with metastatic HER2-positive GEA (Tabernero et al. 2022).

KN026 is another anti-HER2 bispecific antibody that binds non-overlapping epitopes of HER2, leading to dual HER2 signaling blockade and achieving the effect of trastuzumab in combination with patuzumab. A phase II trial (NCT03925974) (Xu et al. 2023) showed that patients with HER2-expressing advanced GC or GEJA receiving KN026 had an ORR of 56% and a durable remission duration of 9.7 months. The phase II KN026-CSP-001 study (NCT05427383) evaluating the efficacy of KN026 in combination with chemotherapy in patients with HER2-positive unresectable advanced or metastatic GC who have failed first-line therapy is ongoing.

An important challenge with bispecific antibodies is the complexity in their development and manufacturing. Bispecific antibodies are designed to simultaneously bind to two different antigens, which can enhance therapeutic efficacy, especially in cancer treatment. However, the engineering of these molecules is technically demanding, requiring precise control over their structure and function (Klein et al. 2024). Additionally, there can be issues associated with stability, solubility, and immunogenicity. The dual-targeting approach may lead to unintended interactions or off-target effects, raising concerns about safety and efficacy (Li et al. 2020a). Furthermore, the regulatory pathway for bispecific antibodies can be more complicated compared to traditional monoclonal antibodies, potentially delaying their approval and availability for patients. Overall, while bispecific antibodies hold great promise, it is crucial to address these challenges for their successful development and clinical application.

Together, ADC such as T-DM1, T-DXd, RC48, and also GQ1001 under development, which is molecularly designed to precisely target and kill cancer cells, has demonstrated remarkable efficacy and good tolerability in GC clinical trials. Bispecific antibodies, such as ZW25 and KN026, bind to different domains of HER2 blocking the corresponding signaling, and have good antitumor activity. The development of these emerging HER2-targeting drugs has opened up new possibilities for GC treatment. However, there remains a way to go to incorporate some of them into the standard treatment of GC, as their antitumor activity and safety still need to be further validated. Future studies need to focus on how to overcome targeted drug resistance associated with tumor heterogeneity to develop more effective and precise therapeutic strategies.

### VEGF/VEGFR-targeting drugs for GC

Angiogenesis involves a series of complex biological processes that are jointly regulated by various angiogenesis-promoting and inhibiting factors. VEGF is one of the most important cytokines in inducing tumor angiogenesis, by promoting the proliferation of endothelial cells and increasing vascular permeability. The expression of VEGF is usually high in GC tissues, and is associated with invasiveness, staging, and prognosis (Xu et al. 2016; Grigore et al. 2013; Li et al. 2017a). The VEGF family includes VEGF-A/B/C/D/E and placental growth factor, with receptors including VEGFR-1/2/3. VEGFR is a RTK consisting of seven immunoglobulin homologous domains that transduce growth factor signaling. Binding of VEGF to VEGFR activates the VEGFR signaling pathway, which regulates biological processes including tumor angiogenesis. Therefore, anti-VEGFR antibodies and VEGF inhibitors are expected to block angiogenesis, thereby reducing tumor blood flow and nutrient supply, increasing vascular permeability, and facilitating drug penetration into the tumor.

Bevacizumab is a humanized IgG1 mAb that blocks the binding of VEGF to VEGFR, thereby blocking activation of the tyrosine kinase signaling pathway. Bevacizumab highly specifically recognizes and binds to VEGF, and is the first FDA-approved anti-VEGF mAb for use in cancer therapy (Xu et al. 2016). The phase III AVAGAST trial on patients with advanced GC showed no significant difference in OS between the first-line bevacizumab combined with chemotherapy and placebo combined with chemotherapy groups (12.1 vs 10.1 months). However, there was a significant increase in PFS and overall remission rate in the bevacizumab combination group (Ohtsu et al. 2011). A pilot study (Yu et al. 2022) showed that patients with locally advanced GC receiving neoadjuvant bevacizumab in combination with chemotherapy had an objective remission rate of 55.0% and a disease control rate of 95.0%.

Ramucirumab is a fully-humanized mAb targeting VEGFR, with specificity for VEGFR2. The phase III REGARD trial (Fuchs et al. 2014) on patients with advanced GC showed that the median OS of the ramucirumab group was prolonged by 1.4 months compared to that of the placebo group, with essentially similar incidence of adverse events, except that the incidence of hypertension was 8% higher in the ramucirumab group. The phase III RAINBOW trial showed that ramucirumab in combination with paclitaxel increased OS by 2.2 months compared to paclitaxel in patients previously treated for advanced GC, with noticeable major adverse effects including leukopenia, neutropenia, hypertension, and decreased strength (Wilke et al. 2014). Thus, ramucirumab in combination with paclitaxel significantly improves OS and can be considered a new standard second-line treatment for patients with advanced GC. However, a phase II trial (Yoon et al. 2016) with ramucirumab added to mFOLFOX6 as first-line treatment for advanced GC and the phase III RAIN trial with ramucirumab added to first-line chemotherapy showed no significant improvement in OS (Fuchs et al. 2019), currently not supporting ramucirumab as first-line treatment of GC or GEJA. Apatinib is a small-molecule TKI targeting VEGFR-2. A phase III trial (Li et al. 2016) in China showed that apatinib treatment significantly improved OS and PFS compared with placebo in patients with advanced GC or GEJA refractory to prior chemotherapy. Although VEGFR-targeted drugs have shown some efficacy in the treatment of GC, their use as first-line therapy requires further study.

Together, targeting angiogenesis is an important anticancer treatment strategy, and anti-VEGF/VEGFR drugs have been used with some success. Clinical trials have shown that bevacizumab or ramucirumab in combination with chemotherapy effectively prolongs the survival of patients with advanced GC. However, the efficacy of anti-VEGF drugs as first-line treatment is not obvious. In addition to antibody drugs, apatinib, a small molecule TKI targeting VEGFR-2, has shown some efficacy in patients with advanced GC. Future studies are needed to further explore in depth markers precisely predictive of efficacy to provide more effective treatment for GC patients.

### Claudin 18.2-targeting therapy for GC

Claudin 18.2 (CLDN18.2) is an isoform of the tight junction protein claudin 18, which is a highly selective biomarker with limited expression in normal tissues, and is often aberrantly expressed during the development of a variety of cancers, including GC (Li et al. 2020b). The phase II FAST randomized trial showed significant improvement in OS and PFS in patients with CLDN18.2-positive advanced GC/GEJA who received zolbetuximab in combination with chemotherapy versus chemotherapy alone (Sahin et al. 2021). The phase III SPOTLIGHT trial similarly found that zolbetuximab combined with folinic acid, fluorouracil, and oxaliplatin (FOLFOX) improved median PFS and OS compared with FOLFOX alone in patients with advanced GC/GEJA (Shitara et al. 2023a).

Currently, an important and major challenge regarding Claudin 18.2-targeting therapy for GC is the potential for off-target effects and the specificity of the therapy. While Claudin 18.2 is expressed in GC cells, it can also be detected in normal tissues, particularly in the stomach (Nakayama et al. 2024). This raises concerns regarding the risks of damaging healthy cells and causing adverse effects. In addition, there may be variability in Claudin 18.2 expression among different patients or even within different tumor regions, which can affect the efficacy of targeted therapies. According to a biomarker analysis of samples from the phase III SPOTLIGHT and GLOW trials, the percentage of patients with Claudin 18.2-positive GC in mainland China (35.0%) is similar to that in North America (37.7%), but lower than that in Europe and the Middle East (44.0%) (Shitara et al. 2023b). Identifying patients who will benefit most from Claudin 18.2-targeting therapies is crucial, but this requires reliable biomarkers and diagnostic tools. Furthermore, resistance mechanisms may develop over time, limiting the long-term effectiveness of such therapies. Addressing these challenges is essential to improve the safety and efficacy of Claudin 18.2-targeting approaches in GC treatment.

### Preclinical targets for GC treatment

With the continuous exploration of the molecular landscape of GC, new potential molecular targets are being gradually uncovered and verified. Mucins, fibroblast growth factor receptors (FGFR), and hepatocyte growth factor receptors (HGFR) serve as emerging potential targets for effective treatment of advanced GC.

#### Mucin-targeting therapy for GC

Mucins are a class of high molecular weight glycoproteins that are widely expressed in vivo (Behera et al. 2015), and are a huge target for the treatment of gastrointestinal cancers (Sun et al. 2023). Mucins are classified into secreted mucins (MUC2, MUC5AC, MUC5B, MUC6, MUC7, and MUC19) and membrane-bound mucins (MUC1, MUC3A, MUC3B, MUC4, MUC12, MUC13, MUC15, MUC16, MUC17, and MUC20) (Sun et al. 2023; Brassard et al. 2022; Bose and Mukherjee 2020). Secreted mucins act as a protective barrier for underlying mucosal cells, whereas membrane-bound mucins play vital roles in cell signaling pathways and cellular interactions. Both types of mucins have highly glycosylated protein cores with multiple tandem repeat sequences rich in proline, threonine, and/or serine; they are also known as PTS structural domains, which have large and dense O-glycan chain structures, and the O-glycans on mucins are collectively referred to as mucin glycans (Sun et al. 2023). Under physiological conditions, mucin glycans are involved in the composition of the mucus barrier, which protects the body from infection and injury. Aberrant expression of mucin glycans can lead to cancer initiation and development (Sun et al. 2023). Mucins are frequently overexpressed in cancers, and targeting mucin glycans for cancer diagnosis and treatment has been promising.

Potentially druggable mucin targets include MUC1, MUC6, and MUC17. Antibodies, radiopharmaceuticals, vaccines, and chimeric antigen receptor (CAR)-T cell therapies have been developed against tumor-associated MUC1 (Sun et al. 2023). Among mucins, the transmembrane glycoprotein MUC1 is the most likely targetable for antibodies against GC, due to its abnormal glycosylation and specific overexpression in cancers (Bose and Mukherjee 2020). The anticancer effects of the MUC1-targeting antibody TAB004 have been demonstrated both in vitro and in vivo (Bose et al. 2023). TAB004 significantly overcomes anoikis-resistance in cancer, and could be potentially used as a prophylactic agent to curb postsurgical cancer relapse, prevent metastasis, and enhance chemotherapeutic efficacy (Bose et al. 2023). Impaired glycosylation of gastric MUC6 or *MUC6* deletion importantly drives spontaneous GC genesis and development and serves as a novel promising therapeutic target (Arai et al. 2024). MUC17 is overexpressed in GC cells and has limited expression in normal cells, and targeting MUC17 for the treatment of GC is currently under investigation (Bailis et al. 2020). Notably, three different types of glycoepitopes (glycan-only, glycopeptide, and shielded-peptide) expressed on tumor-specific MUC1, MUC5AC, and MUC16 can be recognized by monoclonal antibodies (mAb) and may serve as promising candidate target for anticancer ADC development (Brassard et al. 2022). Despite the fact that mucins are important potential targets for GC therapy, most mucin-targeting therapeutic trials have failed to be translated into the clinic, partly due to the complex relationship between different mucins and the lack of sensitive and specific tools for most mucin detections.

#### FGFR-targeting therapy for GC

Alterations in the *FGFR* gene are found in a wide range of cancer entities, with a frequency range of 3–7% in GC/GEJA, and *FGFR1* mutation, *FGFR2* amplification, and *FGFR3* rearrangement are the most common *FGFR* alterations in GC (Helsten et al. 2016). In preclinical models, *FGFR2* amplification predicts the efficacy of the multi TKI regorafenib, whose targets also include FGFR2 (Hur et al. 2020). Currently, several types of FGFR-targeting drugs have been explored or developed for GC, including multi-kinase inhibitors, pan-FGFR inhibitors, FGFR1-3 inhibitors, selective FGFR inhibitors, and ADC (Guan et al. 2023). However, most of the relevant studies are preclinical or single case reports (Yue et al. 2021). A phase I trial showed promising clinical data on the safety, pharmacokinetics, and preliminary activity of bemarituzumab in patients with FGFR2b-overexpressing adenocarcinoma of the stomach or GEJA (Catenacci et al. 2020). The phase II FIGHT trial showed that bemarituzumab plus FOLFOX chemotherapy was generally well-tolerated as first-line treatment in patients with unresectable advanced GC/GEJA with FGFR2b overexpression, but was associated with a higher incidence of adverse events, particularly ocular toxicity, compared with the chemotherapy control (Catenacci et al. 2021).

#### HGFR-targeting therapy for GC

Mesenchymal-epithelial transforming factor (MET), often referred to as c-Met or HGFR, is a receptor tyrosine kinase (RTK) for which HGF is a common ligand (Raj et al. 2022). HGF/MET pathway activation is associated with tumor aggressiveness and poor disease prognosis. Currently, several selective or nonselective c-MET TKI, such as rilotumumab, tinvatinib, AMG 337, and foretinib, are tested in MET-positive GC, but no significant benefits have been observed in clinical trials yet (Catenacci et al. 2017; Kang et al. 2014b; Hong et al. 2019; Shah et al. 2013). Key factors leading to the disappointing results may be the heterogeneity of the target expression in GC and the difficulty in reliably measuring MET overexpression/activation.

## Immunotherapies for GC

With the limited effect of traditional therapy on advanced GC, tumor immunotherapy has emerged as a novel therapeutic strategy that has attracted much attention. Immunotherapies for advanced GC include immune checkpoint inhibitors (ICI), adoptive immune cell therapy, tumor vaccines, oncolytic viruses, and non-specific immunomodulators therapy.

### ICI

Immune Evasion is an important biological feature of cancer cells, where the immune checkpoint pathway is the main mechanism (Liu et al. 2023a; Huang et al. 2023b; Zhou et al. 2023). Cancer cells mainly evade surveillance and killing of human immune system by interfering with immune checkpoints including PD-1, programmed cell death ligand-1 (PD-L1), and cytotoxic T lymphocyte-associated antigen-4 (CTLA-4) (Liu et al. 2023b). Currently, common clinically-available ICI includes antibodies targeting PD-1, PD-L1, and CTLA-4 (Table 2).

**Table 2:** Clinical trials of immune checkpoint inhibitors

Type	Drugs	Clinical trials	Research objects	Regimens	Conclusions of major findings	Adverse event (AE)
PD-1-targeting inhibitors	Nivolumab	Phase III trial Checkmate-649: NCT02872116 (Janjigian et al. 2021a)	AGC, GEJA, and esophageal adenocarcinoma	Nivolumab + chemotherapy vs chemotherapy alone	Combination chemotherapy with nivolumab significantly improved OS and PFS compared to chemotherapy alone	Most common AEs (≥ 25%): nausea, diarrhea, and peripheral neuropathy
	Pembrolizumab	Phase II trial KEYNOTE059: NCT02335411 (Fuchs et al. 2018)	Previously treated AGC and GEJA	Pembrolizumab	Pembrolizumab showed favorable activity and manageable safety profile	Most common AEs: hypothyroidism (8.9%), hyperthyroidism (3.5%), and colitis (2.3%)
		Phase II trial KEYNOTE158: NCT02628067 (Marabelle et al. 2020)	Previously treated recurrent or metastatic solid tumors	Pembrolizumab	Pembrolizumab as second-line treatment of MSI-H/dMMR or TMB-H gastroesophageal cancers was feasible	Grade 4 AEs: colitis
		Phase III trial KEYNOTE062: NCT02494583 (Shitara et al. 2020b)	Untreated advanced GC or GEJA	Pembrolizumab vs pembrolizumab + chemotherapy vs chemotherapy	Pembrolizumab or pembrolizumab combined with chemotherapy did not significantly provide superior OS or PFS	Most common grade 3–5 AEs: anemia, diarrhea, and neutropenia
PD-L1-targeting inhibitors	Atezolizumab	Phase II trial PANDA: NCT03448835 (Verschoor et al. 2024)	Resectable nonmetastatic GC or GEJA	One cycle of atezolizumab monotherapy, four cycles of atezolizumab + docetaxel + oxaliplatin + capecitabine	Atezolizumab combined with chemotherapy improved pathological remission rate in resectable nonmetastatic GC or GEJA	Grade 3 AEs: hepatitis, headache, and diarrhea
	Avelumab	Phase Ib trial JAVELIN: NCT01772004 (Chung et al. 2019)	AGC or GEJA	Avelumab	Avelumab demonstrated clinical activity and an acceptable safety profile in GC or GEJA	Most common AEs: fatigue (10.0%) and nausea (6.7%)
		Phase I trial JPN (Doi et al. 2019)	AGC or GEJA	Avelumab	Avelumab demonstrated acceptable clinical activity in AGC or GEJA and in cancers which progressed following chemotherapy	Most common AEs: pruritus (15.0%), fever (12.5%), and rash (10.0%)
		Phase III trial JAVELIN Gastric 100: NCT02625610 (Moehler et al. 2021)	AGC or GEJA	Avelumab vs chemotherapy (oxaliplatin plus fluoropyrimissdine)	Avelumab did not show significantly better OS compared to continued chemotherapy	Grade 3 + AEs: avelumab, elevated amylase, elevated lipase, weakness, colitis, decreased appetite, hypertension, and pneumonitis; chemotherapy, neutropenia, decreased neutrophil count, and peripheral sensory neuropathy
	Durvalumab	Phase Ib/II trial (Kelly et al. 2020)	AGC or GEJA	Second-line: durvalumab + tremelimumab vs durvalumab or tremelimumab; third-line: durvalumab + tremelimumab	Remission rates were low regardless of whether monotherapy or combination strategy was used	Most common AEs: diarrhea, fatigue, and decreased appetite
		Phase II trial PRODIGE 59-FFCD 1707-DURIGAST: NCT03959293 (Tougeron et al. 2024)	AGC or GEJA	FOLFIRI + durvalumab vs FOLFIRI + durvalumab + tremelimumab	Immune checkpoint inhibitors in combination with FOLFIRI as second-line treatment of AGC or GEJA demonstrated an acceptable safety profile	Most common grade 3–4 AEs: weakness, neutropenia, anemia, and diarrhea
CTLA-4-targeting inhibitors	Ipilimumab	Phase II trial (Bang et al. 2017)	Advanced or metastatic GC or GEJA	Ipilimumab vs best supportive care (BSC)	Overall efficacy of ipilimumab monotherapy was not satisfactory and did not significantly improve immune-related PFS	Most common grade 3–4 AEs: diarrhea and fatigue
		Phase I/II trial CheckMate-032: NCT01928394 (Janjigian et al. 2018)	Metastatic esophagogastric cancer	Nivolumab vs nivolumab + ipilimumab	Nivolumab and nivolumab + ipilimumab provided clinically meaningful antitumor activity in chemotherapy-refractory esophagogastric cancer	Most common grade 3–4 AEs: diarrhea, fatigue, and elevated ALT and AST

*AGC* advanced gastric cancer, *GEJA* gastroesophageal junction adenocarcinoma, *OS* overall survival, *PFS* progression-free survival, *AE* adverse event

#### PD-1 inhibitors

PD-1 is a crucial immune checkpoint receptor on activated T cells and is a member of the co-stimulatory receptor B7/CD28 family. Cancer cells can express PD-L1/2, which inhibit T cell activity when binding to PD-1 on T cells, making cancer cells evade immune surveillance and killing (Keir et al. 2008). Inhibitors targeting PD-1 can bind to PD-1 on the surface of T cells thereby preventing it from binding to PD-L1/2 by cancer cells, which restores T cell activity of killing cancer cells. Common PD-1 inhibitors include nivolumab and pembrolizumab.

Nivolumab is a humanized IgG4 mAb that targets PD-1 and disrupts its interaction with PD-L1/2, thereby increasing the anticancer activity of T lymphocytes. The phase III Checkmate-649 trial showed that for patients with GC and GEJA/esophageal adenocarcinoma, first-line nivolumab combined with chemotherapy significantly improved OS (HR = 0.71, *P* < 0.001) and PFS (HR = 0.71, *P* < 0.001) compared to chemotherapy alone (Janjigian et al. 2021a). Based on the Checkmate-649 trial, in April 2021 the FDA approved nivolumab in combination with fluoropyrimidine- and platinum-based chemotherapy as first-line treatment of patients with advanced or metastatic GC.

Pembrolizumab is also a humanized IgG4 mAb that binds to PD-1 and prevents PD-1 from interacting with PD-L1/2. The phase II KEYNOTE059 trial (Fuchs et al. 2018) of pembrolizumab monotherapy for previously-treated GC and GEJA showed an objective remission rate (ORR) of 11.6% and a median remission duration of 8.4 months, with a manageable safety profile. Based on this trial, in September 2017, the FDA approved pembrolizumab as third-line treatment for PD-L1-positive advanced GC with CPS ≥ 1. The phase II KEYNOTE158 trial (Marabelle et al. 2020) of pembrolizumab for previously-treated advanced solid cancers showed an objective remission rate of 29% and a complete remission rate of 4%, and suggested that pembrolizumab could be used as second-line treatment of MSI-H/dMMR or TMB-H gastroesophageal cancers. In the KEYNOTE062 clinical trial for advanced GC, first-line pembrolizumab or pembrolizumab in combination with chemotherapy was not superior to chemotherapy in terms of OS or PFS (Shitara et al. 2020b). However, in the KEYNOTE059/158/062 studies pembrolizumab alone or in combination with chemotherapy contributed to a higher ORR, median OS, and median PFS in patients with dMMR/MSI-H GC. The 2022 CSCO guidelines recommend first-line pembrolizumab for patients with dMMR/MSI-H GC.

In addition, targeted drugs combined with PD-1 mAb in the first- and second-line setting for GC have demonstrated better therapeutic potential. Based on the phase III Keynote-811 trial (Janjigian et al. 2021b), the FDA has approved pembrolizumab and trastuzumab plus chemotherapy as first-line therapy for patients with HER2-positive irremovable advanced GC/GEJA. The phase Ib REGONIVO trial (Fukuoka et al. 2020) showed that in patients with GC receiving nivolumab combined with regorafenib had an ORR of up to 44.0% and a mPFS of 5.6 months. However, at present, the OS and ORR associated with PD-1 mAb alone or in combination for GC remain low, and how to select the best combination with synergistically-enhanced efficacy deserves further in-depth exploration. We previously reported that the efficacy of PD-1 antibody-based immunotherapy could be well predicted by immune cells (especially neutrophils) in both circulation and the tumor microenvironment (Zhou et al. 2023).

#### PD-L1 inhibitors

PD-L1 belongs to the B7 superfamily of co-stimulatory molecules on antigen-presenting cells and can act as a suppressor of T lymphocytes. Antibodies targeting PD-L1 can attenuate the inhibitory effect of PD-L1 on cytotoxic T cells, thereby accelerating the killing of cancers cells by T cells (Qing et al. 2015). Common inhibitors targeting PD-L1 include atezolizumab, avelumab, and durvalumab.

Atezolizumab is a humanized IgG1 mAb against PD-L1 that reactivates the anticancer immune response by directly binding to PD-L1 and preventing its interaction with PD-1. In 2013, a phase I study (Herbst et al. 2013) reported for the first time the effectiveness of atezolizumab for GC. The phase II PANDA trial (Verschoor et al. 2024) showed that atezolizumab combined with chemotherapy was safe in patients with resectable non-metastatic GC or GEJA, with increased rate of pathological remission. A phase II/III study (NCT03421288) evaluating the efficacy, safety, and hyperimmune reactivity associated with atezolizumab plus FLOT (5-fluorouracil + calciumfolinat + oxaliplatin + docetaxel) versus FLOT alone for patients with GC/GEJA and is ongoing. Another ongoing phase II trial (NCT04661150) is evaluating the efficacy and safety of atezolizumab and trastuzumab in combination with capecitabine and oxaliplatin (XELOX) for patients with HER2-positive resectable locally-advanced GC/GEJA.

Avelumab is also a humanized anti-PD-L1 IgG1 antibody that binds PD-L1 and inhibits its interaction with PD-1. In 2019, two phase I trials (Chung et al. 2019; Doi et al. 2019) showed that avelumab has an acceptable safety profile and good clinical efficacy in patients with advanced GC and GEJA. The phase III JAVELIN Gastric 100 trial (Moehler et al. 2019) showed no significant OS benefits with first-line avelumab maintenance therapy compared with continued chemotherapy in patients with HER2-negative GC. The phase III JAVELIN Gastric 100 trial (Moehler et al. 2021) similarly did not find that avelumab demonstrated a superior mOS over chemotherapy in patients with metastatic GC or GEJA who did not progress after first-line chemotherapy (FOLFOX or XELOX). The phase II MONEO trial (NCT03979131) evaluating the efficacy and safety of perioperative avelumab in combination with chemotherapy for patients with resectable GC or GEJA is ongoing.

Durvalumab recognizes and attaches to the PD-1 receptor on T cells, and it competes with the secreted PD-L1/2 of cancer cells for attachment to the PD-1 receptor on T cells, thus preventing the cancer cells from shutting down the T cells through an immune escape mechanism; this enhances the ability of the immune system to destroy cancer cells. A phase Ib/II trial (Kelly et al. 2020) showed very low response rates with durvalumab alone or durvalumab in combination with tremelimumab for advanced GC or GEJA. However, durvalumab in combination with chemotherapy or durvalumab and tremelimumab in combination with chemotherapy showed significant efficacy as second- and third-line treatment (Tougeron et al. 2024).

#### CTLA-4 inhibitors

CTLA-4 is capable of negatively regulating the immune system and was the first T-lymphocyte ICI to be used clinically. The CTLA-4 mAb binds specifically to CTLA-4 and prevents CTLA-4 from binding to B7, thereby blocking the CTLA-4 pathway and activating T-lymphocytes to kill cancer cells (Kwak et al. 2020). Common inhibitors against CTLA-4 include ipilimumab and tremelimumab. Clinical trials related to the combination of ICI (i.e., anti-PD1/PD-L1/CTLA-4) with chemotherapy have also become a hot research topic.

Ipilimumab is a recombinant humanized IgG1 mAb that binds to CTLA-4, a down-regulator of the T cell activation pathway. Ipilimumab blocks CTLA-4 enhancing T cell proliferation and activation to kill cancer cells. A phase II trial (Bang et al. 2017) showed that the overall efficacy of ipilimumab monotherapy was not satisfactory and did not improve PFS in advanced GC. The phase I/II CheckMate-032 trial showed that nivolumab or nivolumab in combination with ipilimumab showed clinically meaningful anticancer activity in western patients with chemotherapy-refractory esophagogastric cancer (Janjigian et al. 2018).

Tremelimumab is a fully humanized anti-CTLA-4 mAb which blocks the signaling pathway that helps cancers evade immune scrutiny. It kills cancer cells by binding to CTLA-4 on the surface of activated T lymphocytes and stimulating body immune system to launch an attack on cancer cells. Tremelimumab alone or in combination with durvalumab is not effective in advanced GC or GEJA (Kelly et al. 2020), but tremelimumab in combination with durvalumab and chemotherapy is effective in the second- and third-line setting for advanced GC and GEJA (Tougeron et al. 2024).

Together, with the continued research, ICI including PD-1/PD-L1 and CTLA-4 inhibitors has achieved some success and been proved to be effective in the treatment of progressive GC or GEJA, but several important challenges remain. The efficacy of single agent monotherapy remains generally low, and the applicable population remain limited. Future research should focus on combination of drugs as well as identifying markers to screen the advantageous group benefiting more from immunotherapy with optimized regimen.

### Adoptive immune cell therapy

Adoptive immune cell therapy refers to the infusion of ex vivo expanded immune effector cells back into the cancer patient’s body to directly or indirectly stimulate the body’s immune response so as to kill cancer cells achieving the purpose of cancer treatment. According to its targeting nature, immune cell therapies include two categories: targeted therapies, including CAR-T, T cell receptor-engineered T (TCR-T) and CAR-natural killer (CAR-NK) cell therapies, and non-targeted therapies, including cytokine-induced killer cell (CIK) and tumor-infiltrating lymphocyte (TIL) therapies.

#### CAR-T therapy

CAR-T therapy utilizes gene engineering technology to add (chimerize) a receptor for a specific antigen to normal T cells in order to enable the T cells to exert specific killing function. If the antigen is on the surface of a cancer cell, the T cell can specifically bind to the cancer cell, thus killing the cancer cell. Currently, CAR-T therapy targeting CLDN18.2 for advanced GC is most focused. An interim analysis of a phase I trial (Qi et al. 2022) showed that in advanced GI cancers CLDN18.2-targeting CAR-T cell therapy was safe and efficacious, with a 6-month OS rate of 81.2% and an overall remission rate of 57.1%. Due to the nice efficacy, the US FDA approved CLDN18.2 CAR-T for the treatment of GC/GEJA in 2020. However, due to issues associated with the heterogeneity of tumor antigens, tumor infiltration, and the proliferation and stability of T cells within the tumor, CAR-T cell therapy has limited efficacy in GC (Tian et al. 2020; Martinez and Moon 2019). It is important to find new potential therapeutic targets.

#### TCR-T therapy

TCR-T immunotherapy involves transducing the TCR gene, which encodes protein specifically recognizing tumor antigens, into patient's peripheral blood T cells, and modifying the TCR gene of the T lymphocytes or causing T cells to artificially express engineered antigen receptors. This increases the T lymphocyte's ability to specifically recognize and bind to cancer cells. The pool of targeted antigens for TCR-T cell therapy is larger than that of CAR-T cell therapy. TCR-T cells recognize epitopes derived from membrane and intracellular proteins and presented by the major histocompatibility complex (MHC), whereas CAR-T cells only target cell surface antigens. However, the cancer-killing effect of TCR-T is MHC-restricted, and the expression of MHC in cancer cells is usually downregulated, thus limiting the efficacy (Baulu et al. 2023). The application of TCR-T for GC is being investigated. Two phase I trials (NCT05483491 and NCT05035407) investigating the efficacy of TCR-T cell therapies targeting KK-LC-1 for cancers including GC are ongoing in the US.

#### CAR-NK therapy

Nature killer (NK) cells are an important component of the human innate immune system. NK cells can secrete a large number of cytokines to play an immunomodulatory role, and highly express the FcγIII receptor, which can bind to the Fc segment of the tumor antigen-specific antibody, causing antibody-dependent cell-mediated cytotoxicity (ADCC) to directly kill the target cells (Huang et al. 2023b). Unlike T and B cells, NK cells do not need to be pre-sensitized to have killing activity. CAR-NK cell therapy is a CAR genetic engineering modification of NK cells, connecting molecules that recognize specific antigens on the surface of tumors with signaling molecules needed to activate immune cells, breaking through the limitations of inhibitory receptors and activating NK cells; this further enhances the ability of NK cells regarding target recognition and specific killing of tumor cells. Compared with CAR-T, CAR-NK has several advantages: CAR-NK has more tumor-killing pathways; allogeneic use will not cause graft-versus-host disease, and a generic CAR-NK can be generated; CAR-NK does not secrete inflammatory factors (e.g., IL-1 and IL-6), and will not induce a cytokine storm; and CAR-NK has a clear advantage in the treatment of solid tumors (Yang et al. 2016; Liu et al. 2021; Hermanson et al. 2016). The use of CAR-NK in GC is being explored in an ongoing phase II trial (NCT04847466) that evaluates the efficacy of irradiated PD-L1 CAR-NK cells in combination with pembrolizumab and N-803 in patients with advanced GC or head and neck cancer.

#### CIK therapy

CIK cells are a heterogeneous group of cells obtained by coculturing human peripheral blood mononuclear cells with various cytokines in vitro. They are characterized by the expressions of CD3 and CD56, which are surface markers shared by T lymphocytes and NK cells. CIK cells possess both the potent cancer-killing activity of T lymphocytes and the non-MHC-restricted cancer killing of NK cells (Zhang et al. 2020). A clinical trial (Li et al. 2017b) showed that adjuvant immunotherapy with autologous CIK prolonged the disease-free survival of postoperative GC patients. Another study (Qiao et al. 2019) evaluating DC-CIK versus S-1 in combination with cisplatin for advanced GC showed that CIK resulted in longer OS and PFS. There remains a lack of high-level clinical evidence to support the use of CIK in GC, and further research is still needed.

#### TIL therapy

TIL is a heterogeneous group of immune cells, including T, B, and NK cells, that accumulate in tumors and their surrounding tissues. Transluminal TIL therapy involves isolating and culturing lymphocytes from around the tumor tissue and infusing them back into the patient. The effect of TIL in solid tumors, including GC, is not yet definitive (Zhang et al. 2019; Sarnaik et al. 2021; Meaningful response to TILs in NSCLC 2021). A phase I/II trial (NCT04426669) evaluating the efficacy of TILs for the treatment of metastatic gastrointestinal cancers is currently ongoing in the US, and another phase II trial (NCT03935893) evaluating the efficacy and safety of autologous TILs in patients with metastatic solid cancers is ongoing in China.

### Tumor vaccines

Tumor vaccine therapy is an active immunotherapy that introduces tumor antigens into the patient's body, activates the body's immune system, and enhances the immune response to tumor, and is well-tolerated with little to no dose-related toxicity (Saxena et al. 2021). Antigens for tumor vaccines are divided into two categories: tumor-associated antigens (TAA) and tumor-specific antigens (TSA). Phase I/II GC vaccine-related trials validated a variety of TAA antigens, all of which demonstrated a favorable safety profile, but the OS of patients was not significantly improved; thus further optimization of efficacy is needed (Kole et al. 2022). TSAs, also known as neoantigens, are a class of peptides expressed on the surface of tumor cells, which can stimulate T-cell-mediated cytotoxic antitumor immune response, thus causing T-cell population expansion.

Common GC vaccines include protein/peptide vaccines, gene vaccines, cellular vaccines, and genetically-engineered vaccines. Protein/peptide vaccines are prepared using antigenic peptides eluted from the surface of tumor cells or proteins abnormally expressed inside tumor cells, which have the advantages of high specificity and safety. A phase I/Ib trial (NCT01227772) (Sundar et al. 2018) showed that in patients with advanced GC the OTSGC-A24 combined peptide cancer vaccine was well-tolerated. Gene vaccines (including DNA and RNA vaccines) have the advantages of being easy to produce, applicable to a wide range of diseases, and inexpensive to store under freeze-dry conditions. A clinical trial (NCT03468244) is currently investigating the efficacy of mRNA vaccines in GC, with promising preliminary findings. The main types of cellular vaccines related to GC are dendritic cell (DC) vaccines and cancer stem cell (CSC) vaccines. DCs are the main cells presenting antigens in the body and play a very important role in the tumor-specific immune response. DC vaccines are made by obtaining the patient's own DCs, cultivating them in vitro, and then inoculating them into the patient. Currently, there are four DC tumor vaccines approved and marketed globally, but there is no vaccine for GC (Saxena et al. 2021). The CSC vaccine is also very promising. Commonly used vectors for genetically engineered vaccines are viral and yeast vectors.

Although there are no FDA-approved tumor vaccines for GC treatment on the market, the field has a very promising future, and tumor vaccines including the MG-7-DC vaccine (NCT04567069), the OTSGC-A24 peptide vaccine (NCT03784040), and the peptide vaccine targeting HER2, IMU-131 (HER-Vaxx) (NCT02795988) are being investigated on GC.

### Oncolytic viruses

Oncolytic viruses are a class of natural or recombinant viruses that selectively kill cancer cells without harming normal cells (Javid et al. 2023a). Oncolytic viruses mediate anticancer responses through a dual mechanism, including viral lysis of cancer cells and induction of host-anti-cancer immunity (Kaufman et al. 2015). Preclinical studies have confirmed that oncolytic viruses can enhance anticancer immune response and effectively control tumor growth in GC and colorectal cancer, through direct tumor killing effects (Kana and Essani 2021). We previously conducted a prospective study (Zhang et al. 2023) showing that in patients with liver metastases from gastrointestinal cancers, administration of recombinant human adenovirus type 5 was safe and well-tolerated, and resulted in a disease control rate of 74% after two courses. Also, more clinical evidence is needed to support the use of lysosomal viruses in GC. The most meaningful function of lysosomal viruses is to attract immune cells to the tumor microenvironment (Muscolini et al. 2020), which would significantly improve the efficacy of immunotherapy against GC. Thus, lysosomal viruses can serve as powerful catalysts to assist other treatments, enabling GC patients to benefit more from cancer treatment.

### Nonspecific immunomodulators therapy

Nonspecific immunomodulators, including interleukin (IL), interferon (IFN), and tumor necrosis factor (TNF), treat tumors by stimulating immune effector cells. IL-2 promotes the proliferation of immune cells, induces the production of a variety of cytokines, promotes the proliferation of NK cells, and enhances the body's anticancer ability. Several IL-2-based second-generation compounds in combination with the ICI atezolizumab (NCT03138889), nivolumab (NCT02983045, NCT03282344, and NCT03435640), and nivolumab plus ipilimumab (NCT02983045) are in clinical trials (Berraondo et al. 2019). IFN is an active protein with multiple functions which is produced by monocytes and lymphocytes. IFN-α has broad-spectrum antiviral and immunomodulatory effects, and can also activate the immune system to exert anticancer effects (Cauwels et al. 2018). TNF is a small molecule protein secreted by macrophages and T-lymphocytes that kills or inhibits cancer cells, promotes inflammatory responses, and regulates the immune system and angiogenesis. The use of IFN-α and TNF in GC therapy is still under investigation.

Currently, an important and major challenge with nonspecific immunomodulator therapies for GC is the risks of systemic adverse effects and toxicity (Berraondo et al. 2019). Since these therapies aim to broadly enhance the immune response, they can inadvertently activate immune cells against healthy tissues, leading to autoimmune reactions and/or inflammatory adverse effects. This can result in a range of complications, including skin rashes, gastrointestinal issues, and even damages to vital organs. In addition, the variability in individual patient responses to nonspecific immunomodulators complicates treatment. Factors such as genetics, existing health conditions, and the tumor microenvironment can influence how well a patient responds to treatment, making it difficult to predict outcomes (Propper and Balkwill 2022). Balancing the therapeutic benefits of enhancing immune function and the associated adverse effects which meanwhile need to be minimized is a significant challenge in the clinical use of nonspecific immunomodulators. Developing more targeted approaches that selectively enhance immune responses against cancers without affecting healthy tissues is an important ongoing area of research.

## Conclusions, chanllenges, and perspectives

**Fig. 1:**
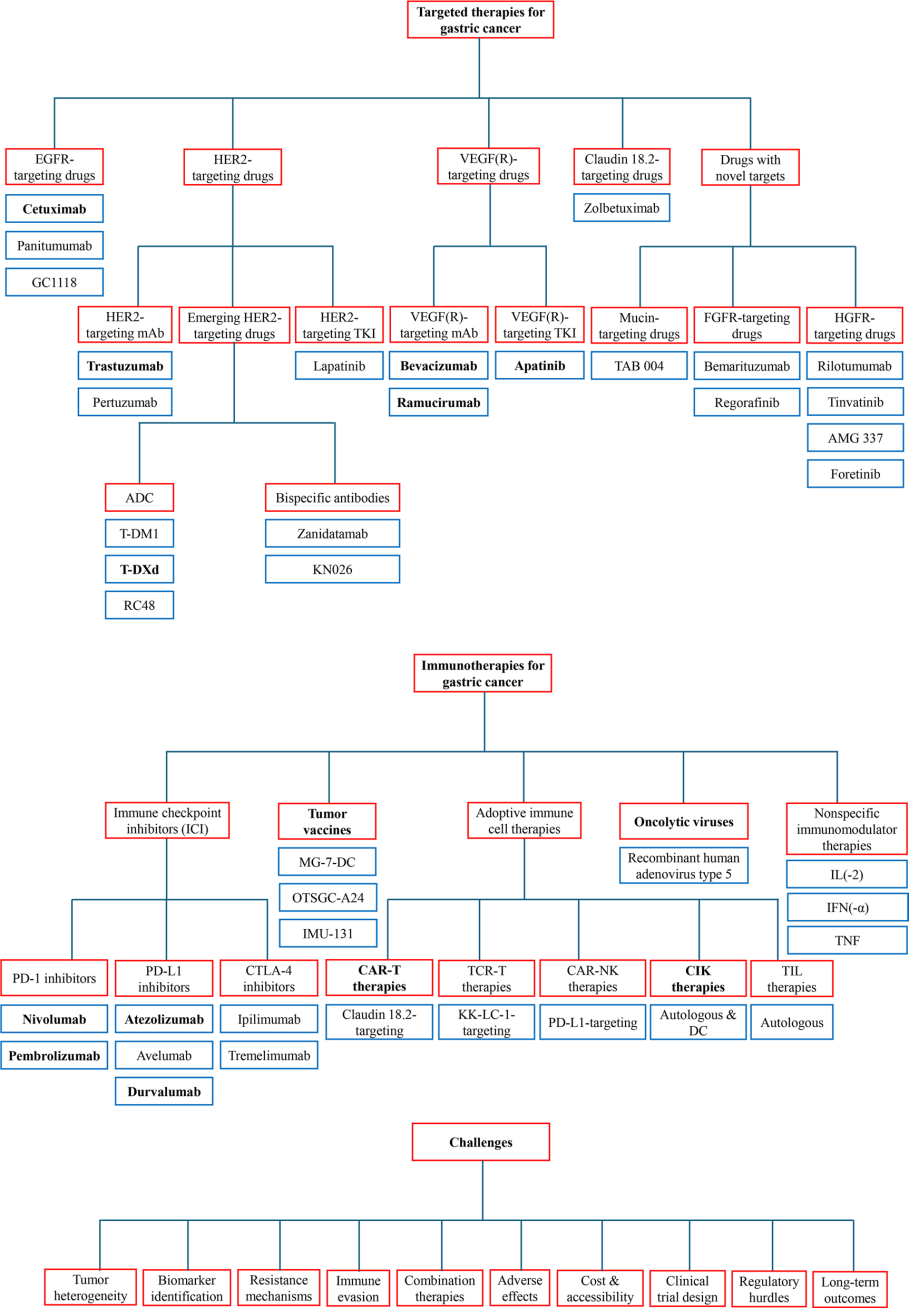
Schematic design diagram showing targets and representative drugs of targeted therapy and immunotherapy for gastric cancer and associated challenges. Drugs and therapies in **bold** indicate those that can be considered for the treatment of gastric cancer (alone or in combination) under current evidence, and the others are being investigated in (pre-)clinical studies or currently with less promising or negative findings (Bang et al. 2010, 2017; Zhang et al. 2014, 2023; Quintero Aldana et al. 2020; Oh et al. 2019; Tabernero et al. 2018; Iqbal et al. 2011; Hecht et al. 2016; Thuss-Patience et al. 2017; Doi et al. 2017, 2019; Shitara et al. 2019, 2020a, b; Cutsem et al. 2021; Xu et al. 2021, 2023; Peng et al. 2021; Meric-Bernstam et al. 2021; Ku et al. 2021; Ohtsu et al. 2011; Fuchs et al. 2014, 2019, 2018; Wilke et al. 2014; Yoon et al. 2016; Li et al. 2016; Janjigian et al. 2021a, 2018; Marabelle et al. 2020; Verschoor et al. 2024; Chung et al. 2019; Moehler et al. 2021; Kelly et al. 2020; Tougeron et al. 2024)

**Fig. 2:**
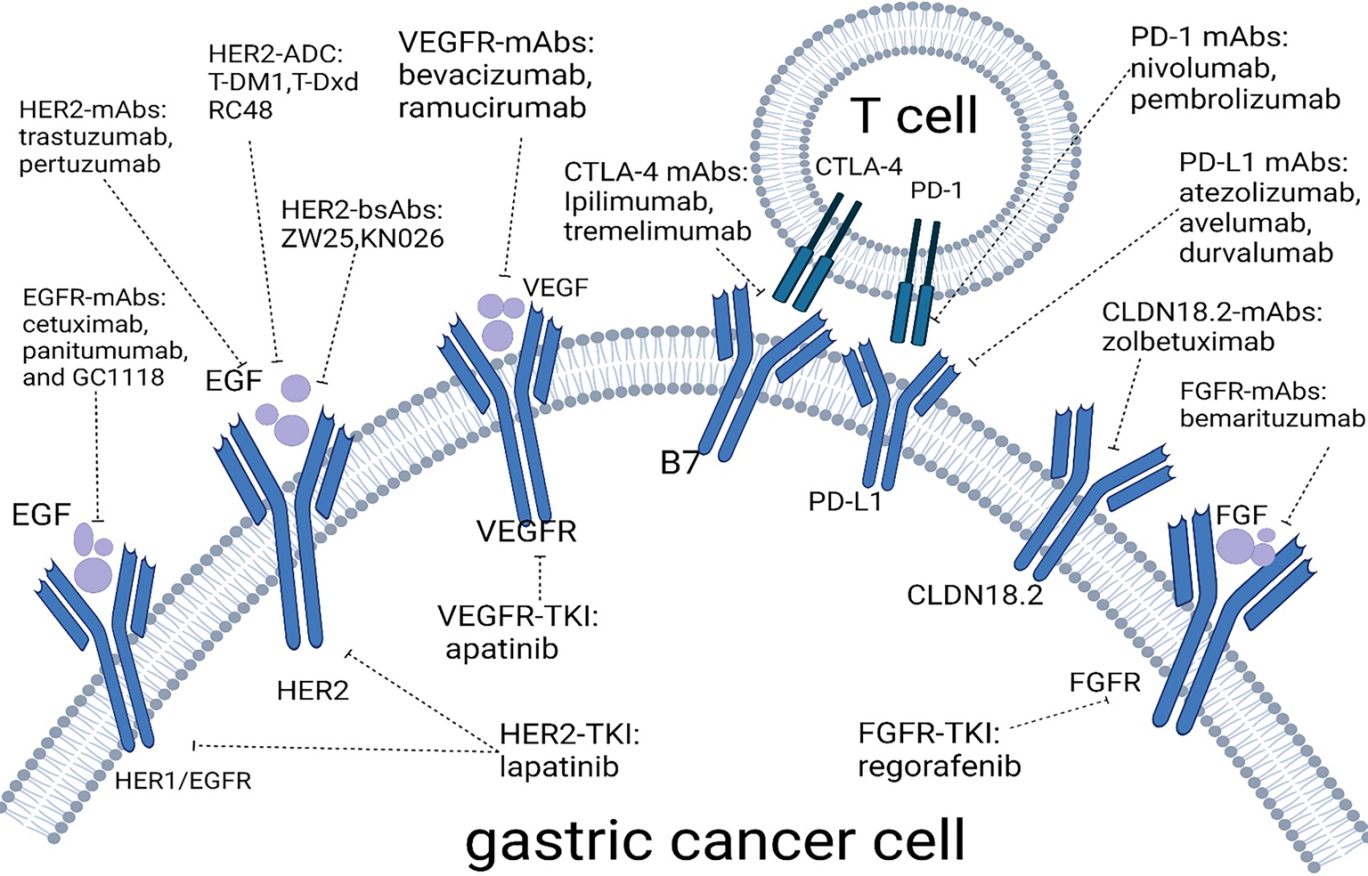
Schematic plot illustrating targets and corresponding drugs of targeted therapy and immunotherapy for gastric cancer (Bang et al. 2010, 2017; Zhang et al. 2014, 2023; Quintero Aldana et al. 2020; Oh et al. 2019; Tabernero et al. 2018; Iqbal et al. 2011; Hecht et al. 2016; Thuss-Patience et al. 2017; Doi et al. 2017, 2019; Shitara et al. 2019, 2020a, b; Cutsem et al. 2021; Xu et al. 2021, 2023; Peng et al. 2021; Meric-Bernstam et al. 2021; Ku et al. 2021; Ohtsu et al. 2011; Fuchs et al. 2014, 2019, 2018; Wilke et al. 2014; Yoon et al. 2016; Li et al. 2016; Janjigian et al. 2021a, 2018; Marabelle et al. 2020; Verschoor et al. 2024; Chung et al. 2019; Moehler et al. 2021; Kelly et al. 2020; Tougeron et al. 2024)

Herein we comprehensively review the current status and advances of the application of targeted therapy and immunotherapy for GC and summarize their effectiveness, highlighting the advantages, limitations, and future directions of such treatments (Figs. 1, 2). Targeted therapy and immunotherapy have brought hope and provided better treatment options for GC patients. This review is on targeted therapy and immunotherapy for GC aiming to personalize treatment based on the specific molecular and genetic characteristics of the tumor. This precision medicine strategy can improve patient outcomes by selecting therapies that are more likely to be effective for individual patients, reducing reliance on traditional chemotherapy, which can have broader and often more severe side effects. By focusing on specific pathways or immune responses, targeted therapies and immunotherapies can potentially lead to better survival outcomes and improved quality of life for patients with GC. Based on current evidence, EGFR-targeted mAb (Cetuximab), HER2-targeted mAb (trastuzumab), HER2-targeted ADC (T-DXd), VEGF/VEGFR-targeted mAb (bevacizumab and ramucirumab [second-line]) and TKI (apatinib [second-line]), PD-1-targeted mAb (nivolumab and pembrolizumab [second/third-line]), PD-L1-targeted mAb (atezolizumab and durvalumab [second/third-line]), immune cell therapies (CAR-T and CIK), tumor vaccines, and oncolytic viruses alone or in combination, can be considered for the treatment of GC and/or GEJA. Future research needs to continue exploring the molecular mechanisms of GC in depth, searching for new therapeutic targets and drugs, and investigating immune escape mechanisms guiding how to overcome drug resistance, in order to further improve the efficacy of GC treatment and bring better survival and quality of life to GC patients.

Despite the progresses in targeted therapy and immunotherapy for GC, there remain some important challenges and pending issues. Notably, many of the targeted therapies and immunotherapies for GC failed, which could be potentially evaded through several approaches (discussed especially in the following first to sixth points). First, GC shows significant genetic and phenotypic heterogeneities (Guan et al. 2023), making it difficult to identify universal targets for treatment. This variability can result in differential responses to targeted drugs and immunotherapies in different patients. The Cancer Genome Atlas project divided GC into four molecular subtypes, which are Epstein-Barr virus-positive, microsatellite unstable, genomically stable, and chromosomally unstable, respectively (Cancer Genome Atlas Research Network 2014). Mismatch repair deficiency makes cancers highly sensitive to PD-1-inhibiting immune checkpoint blockade (Le et al. 2015; Lee et al. 2016; Jiang et al. 2018). More accurate and reliable predictors are needed to precisely select patients. Individualized treatment strategies based on the molecular characteristics of cancers are warranted to help reduce ineffective treatments and adverse events (Huang et al. 2023c, 2024). Second, reliable biomarkers for appropriate selection of patients are currently still under active investigation and need to be further explored. Not all patients respond or equally respond to treatment, and identification of predictive biomarkers is crucial for maximizing therapeutic efficacy. Although PD-L1 expression has been widely used to predict patient response to ICI, its predictive ability is limited and impacted by patient and cancer heterogeneities, tumor immune microenvironment, sample collection and testing methods, and the dynamic nature of PD-L1 expression (Triantafillidis et al. 2024; Hou et al. 2023; Peixoto et al. 2023). Other potential biomarkers included tumor mutation burden (TMB) (Goodman et al. 2017) and microsatellite instability (MSI) (Pietrantonio et al. 2021). Additional biomarkers should be explored in the future to screen patient subpopulations with high sensitivity to treatment and to optimize therapeutic plans, and single cell sequencing and spatial transcriptomics might facilitate this exploration. It is also important to identify novel therapeutic targets for novel treatment strategies. Third, therapeutic resistance is a major problem with targeted therapy and immunotherapy. Many patients initially respond to targeted therapies or immunotherapies but eventually develop resistance. GC cells acquire therapeutic resistance through multiple mechanisms, including gene mutations, signaling pathway reorganization, and alterations of the tumor microenvironment (Jiang et al. 2018; Huang et al. 2014b, 2015a, b, c; Guo et al. 2021b; Jia et al. 2020; Wu et al. 2017). Investigating and understanding the mechanisms of treatment resistance is vital for developing second-line therapeutic strategies and overcoming this challenge. For instance, while the HER2-targeting T-DXd is highly efficacious against many GC cases, its efficacy could be compromised by ABCG2 expression, which is highly prevalent in GC and which could cause treatment resistance (Li et al. 2018; Nagai et al. 2019; Weng et al. 2023; Zhang et al. 2013). Novel ADC with payloads which are not substrates of ABCG2 might effectively address this resistance. Fourth, GC can develop mechanisms to evade the immune system, which can be divided into three categories: Loss of or alterations in tumor-associated antigen (TAA), damage to the antigen presentation mechanism (APM), and immunosuppression in the tumor microenvironment (TME) (Wang et al. 2022). Tumor cells can modify the TAA and APM, and those that have evaded the anticancer immunity can impact the TME through multiple signaling (e.g., immune checkpoints), resulting in downregulation of antigen presentation (Peng et al. 2017; Ma et al. 2022) and/or secretion of immunosuppressive factors (Salaroglio et al. 2019; Zdanov et al. 2016), which can limit the effectiveness of immunotherapy. More researches and trials should be conducted in the future to better understand mechanisms of immune evasion and explore more inhibitors of immunosuppression. Fifth, the combination of targeted therapies and immunotherapies shows clinical promise (Guan et al. 2023; Upadhaya et al. 2021). For instance, based on the CheckMate 649 trial (Janjigian et al. 2021a), nivolumab plus chemotherapy has been approved as first-line therapy for advanced gastric and gastroesophageal junction adenocarcinomas. Notably, it remains an outstanding challenge to determine the optimal combinations, dosages, and therapeutic schedules. To establish effective protocols, it is critically needed to further design clinical trials to adequately assess the efficacy and safety of new combination therapies in different GC populations with varying cancer characteristics. Sixth, targeted therapies and immunotherapies can cause significant side effects that may affect patient prognosis and quality of life. The side effects of targeted and immunotherapeutic drugs are listed in Tables 1, 2 and 3 (Bang et al. 2010, 2017; Zhang et al. 2014, 2023; Quintero Aldana et al. 2020; Oh et al. 2019; Tabernero et al. 2018; Iqbal et al. 2011; Hecht et al. 2016; Thuss-Patience et al. 2017; Doi et al. 2017, 2019; Shitara et al. 2019, 2020a, b; Cutsem et al. 2021; Xu et al. 2021, 2023; Peng et al. 2021; Meric-Bernstam et al. 2021; Ku et al. 2021; Ohtsu et al. 2011; Fuchs et al. 2014, 2019, 2018; Wilke et al. 2014; Yoon et al. 2016; Li et al. 2016; Janjigian et al. 2021a, 2018; Marabelle et al. 2020; Verschoor et al. 2024; Chung et al. 2019; Moehler et al. 2021; Kelly et al. 2020; Tougeron et al. 2024). Some targeted drugs may trigger adverse events including interstitial lung disease, neurotoxicity, cardiotoxicity, skin reactions, and liver function abnormalities, and immunotherapy might lead to off-target effects and systemic toxicity, and induce tumor hyper-progression, highlighting the need and importance of more comprehensive safety assessment and management. Managing these adverse effects while maintaining treatment efficacy is a critical concern. Seventh, the high costs of targeted therapies and immunotherapies can limit access for many patients with GC, particularly in low-resource settings. Ensuring equitable access to these therapies is also an important ongoing challenge and issue. One possible solution is to increase the health insurance coverage of such therapies. Eighth, to optimize therapeutic efficacy and applicability, it is critically needed but complex to further design clinical trials which adequately assess the efficacy and safety of new therapies in diverse GC populations with different tumor characteristics; these trials should not only take into account the heterogeneities among patients and cancers, but must also be dynamically adapted considering the continuously evolving landscape of therapeutic options (Wang et al. 2024). Nineth, it can also be challenging and complex to navigate the regulatory pathways for the approval of novel targeted and immunotherapeutic agents with regulatory hurdles frequently seen, particularly when it comes to demonstrating their safety and efficacy across diverse patient populations; this important aspect may further limit patient access to advanced treatments. Tenth, currently there remain limited data on the long-term outcomes of GC patients managed with targeted therapies and/or immunotherapies (Guan et al. 2023). Understanding the durability of responses to these therapies and their combinations and the long-term survival benefits associated with such therapies is essential for enhancing clinical practice, which will not only help to improve patient prognosis and quality of life, but also provide clinicians with an important basis for more precise decision-making. To improve outcomes in GC treatment, it requires a multidisciplinary approach involving ongoing research, collaborations among healthcare providers and policymakers, and patient-centered strategies to address these challenges. Eleventh, there are several important immune-oncology challenges of note. Patients may respond poorly to immunotherapy and some patients may not respond or respond with progressive disease, due to the immunosuppressive role of the tumor microenvironment (Javid et al. 2023b; Rastin et al. 2024a). It is also an urgent unmet need to enhance the targeted delivery of immunotherapeutic agents to minimize off-target effects and undesired toxicities. Emerging strategies, such as the use of zeolitic imidazolate framework-8 (ZIF-8) nanoparticles to encapsulate and deliver PD-1 inhibitors in a precise tumor-targeting manner with controlled drug release, can potentially augment the efficacy of cancer immunotherapy, minimize systemic toxicity, and improve patient tolerability and outcomes, striking a balance between efficacy and safety (Rastin et al. 2024b; Oryani et al. 2024). Future studies should continue to explore the integration of targeted delivery systems and immunotherapeutic agents to reduce treatment-related side effects (Javid et al. 2024a, b; Karimi-Shahri et al. 2023).

**Table 3:** Major side effects associated with immunotherapy

Immunotherapy types	Major side effects
Immune checkpoint inhibitors (ICI)	Systemic response: Weariness
	Digestive system symptoms: Nausea, diarrhea, and colitis
	Hematological abnormalities: Anemia and neutropenia
	Liver function abnormalities: Hepatitis
Adoptive immune cell therapies	Hematological abnormalities: Leukopenia, neutropenia, and anemia
	Digestive symptoms: Colitis
	Immune response issues: Cytokine release syndrome (CRS) and autoimmune response
Tumor vaccines	Immune response issues: Foreign body rejection and immune tolerance
	Local reactions: Redness, swelling, and pain
	Systemic reactions: Fever and malaise
Oncolytic viruses	Issues associated with antiviral immunization
	Immune response: Cytokine release syndrome and allergic reaction
	Security issues: Live virus transmission
Nonspecific immunomodulators therapy	Immune response problems: Immune overreaction leading to autoimmune reactions and inflammatory side effects
	Systemic side effects: Rash and gastrointestinal reactions

Future studies should enhance therapeutic efficacy by simultaneously inhibiting multiple key signaling pathways and angiogenesis with multiple targeted agents, with adverse events carefully monitored. Further enhancement of the patient's anticancer immune response could be achieved through immunotherapy in combination with other immunomodulators. Precise and individualized selection of the most efficacious treatment strategy based on tumor genotype, phenotype, and other characteristics is warranted. Identification of new targets and development of novel targeted immunotherapeutic agents with higher efficacy to overcome the problem of drug resistance is also an unmet need. Breakthroughs in these research directions can further improve the outcome of GC patients. In addition, extensive translational research, preclinical studies, and multi-omics-based clinical trials hold promise and will make breakthroughs in the treatment of GC in the future, further improving the OS and quality of life of patients with GC, and enhancing the standard of care for GC patients.

## Data Availability

No datasets were generated or analysed during the current study.
